# Screening and genetic characterization of thermo-tolerant *Synechocystis sp*. PCC6803 strains created by adaptive evolution

**DOI:** 10.1186/1472-6750-14-66

**Published:** 2014-07-17

**Authors:** Ulrich M Tillich, Nick Wolter, Philipp Franke, Ulf Dühring, Marcus Frohme

**Affiliations:** 1 Molecular Biotechnology and Functional Genomics, Technical University of Applied Sciences Wildau, Bahnhofstraße 1, 16-2001, D-15745 Wildau, Germany; 2 Institute of Biology, Humboldt-University Berlin, Berlin, Germany; 3 Algenol Biofuels Germany GmbH, Berlin, Germany

**Keywords:** Cyanobacteria, *Synechocystis*, Thermal tolerance, HTS, NGS, Adaptive evolution

## Abstract

**Background:**

Temperature tolerance is an important aspect for commercial scale outdoor cultivation of microalgae and cyanobacteria. While various genes are known to be related to *Synechocystis* sp. PCC6803's heat shock response, there is very limited published data concerning the specific genes involved in long term thermal tolerance. We have previously used random mutagenesis and adaptive evolution to generate a mixture of strains of *Synechocystis* sp. PCC6803 with significantly increased thermal tolerance. The genetic modifications leading to the phenotypes of the newly generated strains are the focus of this work.

**Results:**

We used a custom screening platform, based on 96-deepwell microplate culturing in an in house designed cultivation chamber integrated in a liquid handling robot for screening and selection; in addition we also used a more conventional system. The increased thermal tolerances of the isolated monoclonal strains were validated in larger bioreactors and their whole genomes sequenced. Comparison of the sequence information to the parental wild type identified various mutations responsible for the enhanced phenotypes. Among the affected genes identified are *clpC*, *pnp*, *pyk2*, *sigF*, *nlpD*, *pyrR*, *pilJ* and *cya1*.

**Conclusions:**

The applied methods (random mutagenesis, in vivo selection, screening, validation, whole genome sequencing) were successfully applied to identify various mutations, some of which are very unlikely to have been identified by other approaches. Several of the identified mutations are found in various strains and (due to their distribution) are likely to have occurred independently. This, coupled with the relatively low number of affected genes underscores the significance of these specific mutations to convey thermal tolerance in *Synechocystis*.

## Background

Microalgae and cyanobacteria could be used for large scale outdoor cultivation, e.g. for the production of biofuels. This has led to a revamped interest in biotechnology of cyanobacteria in recent years [1-4]. Wild-type strains however are not ideal for such artificial growth environments. One possibility to create better production strains is classical strain improvement by adaptive evolution, which is defined as the propagation of advantageous mutations through positive selection. Genome wide random mutagenesis can be used to increase the rate of mutations above its natural level, while *in vivo* selection is used to apply the selective pressure. Herein, with only little knowledge about the underlying molecular biology, significant alterations in the phenotype may be promoted. It is also possible to achieve effects which require simultaneous modifications of many seemingly unrelated genes [5].

One important target for optimization is the growth temperature range, in particular when considering desert areas for outdoor cultivation. In a previous work we demonstrated the use of adaptive evolution to produce a mix of strains of *Synechocystis* sp. PCC6803 – a model organism for cyanobacteria and phototrophs as a whole - with a significantly increased thermal tolerance compared to the parental wild type [6]. However this work was limited to the description of the mutagenesis method itself, as well as the phenotype of the generated mix; no monoclonal strains were isolated and no genetic characterization was performed. The genetic modifications leading to the phenotypes of the newly generated strains are the focus of this work.

With the combination of agents used for the generation of the mutants (Ultraviolet Light (UV) and Methyl-methanesulphonate (MMS)), all kinds of mutations (transitions, transversions, frameshifts, and deletions) are possible, though the mutagens used have varied tendencies towards the induction of different kinds of mutations. UV induces mainly transitions from Guanine/Cytosine to Adenosine/Thymine, while deletions mainly occur in A/T rich regions [7], whereas MMS induces all kinds of base substitutions (transitions and transversions), though mainly transversions from GC to TA and vice versa [8,9]. Introduced mutations can be repaired by specific cellular mechanisms, though the mutagenesis protocols used should minimize their activity [6]. Additionally unspecific, error-prone cellular repair mechanisms (such as the SOS response) occur, which are able to induce any type of indirect mutations [7,10,9,11].

*Synechocystis* is polyploid with the number of genome copies per cell reported to vary from 58 to 218 [12]. Numerous genes are already known to be related to *Synechocystis* heat shock response, such as hrcA [13] and hik34 [14] which regulate the expression of heat shock proteins. The heat shock response however, while related, is distinct from long term temperature tolerance. The knockout of hik34 for example, while strongly increasing expression of heat shock proteins in *Synechocystis*, barely affected its survival at the elevated temperature of 47°C (3 h survived, compared to 2 h for the wild type strain) [14]. Many genes (such as chaperonins and proteases) have been shown to be differentially expressed after short term acclimation to higher temperatures [15,16], however, there is very limited published data concerning the specific genes involved in long term thermal tolerance in *Synechocystis*.

Inoue et al. analyzed the growth of *Synechocystis* at various temperatures and determined a very similar growth rate for temperatures ranging from 25 to 40°C. Above (and below) this temperature the growth rate drops rapidly, with no growth at 45°C [17]. They speculated that cell death at higher temperatures does not occur directly because of photosynthesis inhibition, as is often assumed because of Photosystem II sensitivity to higher temperatures [18,15], but rather because of damage to other cell mechanisms, such as the functions of the plasma membrane [17].

Before the genotype of the various previously generated thermo-tolerant strains can be analyzed to determine which genes were affected by the mutagenesis, they have to be isolated and cultured as individual strains. A screening method to determine the individual temperature tolerances of many clones would allow narrowing the focus towards the best performing strains. However, there are currently no commercial systems available for mid- or high-throughput screening of phototrophic microorganisms such as cyanobacteria. We have previously developed a prototype for such a system with the capability for precise temperature control. Furthermore this prototype has recently been greatly expanded and improved with subsequent adaption to a more modern and larger pipetting platform (unpublished results).

The availability of an annotated genome for *Synechocystis*[19], which can be used as a mapping reference greatly reduces the amount of sequencing-reads needed at any given position for a sufficient coverage. Together with increasingly inexpensive next generation sequencing (NGS) techniques it became feasible to sequence various strains with enhanced temperature tolerance (as well as the parental wild type), to identify genes of interest.

We utilized the 454 GS Junior (Roche) and the Ion Torrent PGM (Life Technologies) bench-top sequencers, both of which have enough throughput for the re-sequencing of bacterial genomes. While their technical implementations differ, in both platforms, insertions or deletions (Indels) in homopolymer regions are the main source of errors [20,21]. This leads to an overall higher error rate compared to the competing MySeq platform (Illumina); however both have higher accuracy when detecting base substitutions at the same coverage [22,23]. Though it has been noted for the IonTorrent, that while the percentage of correctly called true SNPs is higher compared to Illumina’s MySeq, the false positive rate is also increased [24].

Here we use our established screening platform, as well as a more conventional system based on a shaking incubator, to screen for the best strains out of the previously generated mix, i.e. those with the highest thermotolerance. The phenotypes of these strains are then characterized and their genetic makeup analyzed with NGS and Sanger sequencing. Various mutations conferring the increased thermal tolerance to the strains are identified and their possible effects discussed.

## Results

### Wild type reference

To identify new mutations generated after mutagenesis and selection under thermal stress, the parental wild-type strain was sequenced as a baseline for further analysis. The combined sequencing coverage was 19-fold with an average raw accuracy across each individual base position in a read of 99.57% and a median read length of 524 bp. Several changes were found compared to the originally published Kazusa-wild-type:

There were four large deletions detected in the genome; three on the chromosome (1.200.305 – 1.201.486; 2.048.411 – 2.049.595; 3.400.331 – 3.401.515) and one on plasmid pSYSM (117.276 – 118.460). Also various SNPs (Additional file 1: Table S1) were identified. All alterations in the wild type were also present in the sequenced thermo-tolerant strains. Additionally, the plasmids pCC5.2 and pCB2.4 could not be identified; no single read properly mapped unto them, even with separate blast alignment.

### Screening and characterization of temperature tolerant strains

A mix of thermo-tolerant strains (mutIV-mix) was previously generated through random mutagenesis and adaptive evolution [6]. To obtain monoclonal strains dilutions were plated on agar-media and single colonies then picked. It is important to note however that while strains obtained via this method originate from a single ancestral cell and are thus by definition monoclonal, due to the polyploidic character of *Synechocystis* each monoclonal strain could possibly contain various differing variants of genes within the many genome copies per cell.

Initial attempts at isolating random single clones from plate without screening for performance yielded strains without notably increased thermal tolerance compared to the wild type (data not shown). Therefore two distinct screening methods were applied.

The first is based on culturing isolated monoclonal strains in small cell culture flasks within a commercially available shaking incubator (incubator screening). In this screening, out of 27 strains analyzed, 6 demonstrated higher thermal tolerance than the wild type (wt) controls. The second approach is based on an automated high throughput screening system for phototrophic microorganisms within Deepwell plates (robot screening), which was developed in house. This system identified 8 (out of 81) strains with significantly higher thermal tolerance than the parental wild type. None of the control wells containing only media showed any increase in OD, which would have indicated cross contamination.

**Figure 1:**
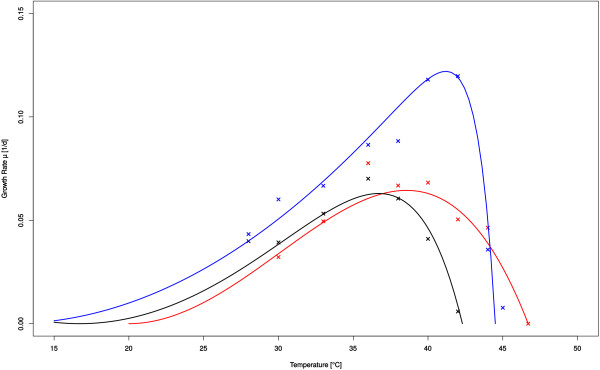
Growth rates over temperature as determined by incubator screening for wt (black), mutIV-mix (red), and C5K (blue).

**Figure 2:**
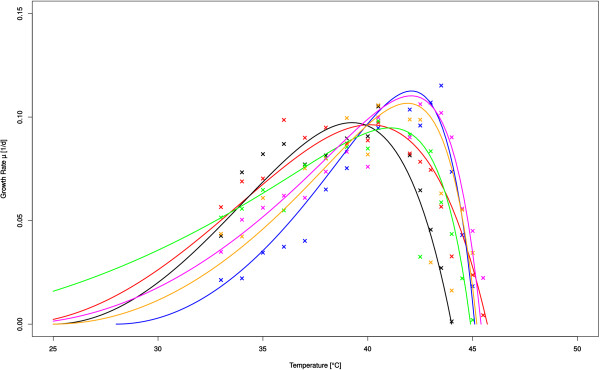
Growth rates over temperature as determined by robot screening: wt (black), mutIV-mix (red), A1 (green), A6 (blue), H2 (magenta), and H12 (orange).

For both approaches the growth rates were determined over a range of temperatures and data then fitted using a modified Ratkowsky-Equation. The data-fitting was not used to determine the maximum tolerated temperature of the strains but merely to allow an easier visualization and comparison of data. In the incubator screening, the strain C5K was identified as having the highest temperature tolerance (Figure 1) and was therefore validated with bioreactor cultivation as well as sequenced. Figure 2 shows the results of the robot screening for four strains (A1, A6, H2, and H12), which were selected for further validation in a 1 l bioreactor and sequencing, since their temperature tolerances were the most promising. In the bioreactor validation the maximum temperature was determined by stepwise increase in temperature until stable growth was no longer possible.

The results for the determined maximally tolerated temperature for the various strains (or mixes) as identified either in the screening systems or the 1 l photobioreactos, as well as the number of mutations identified are summarized in Table 1.

**Table 1:** Overview of the different maximally tolerated temperatures of the various strains in both screening platforms, and in the 1 l bioreactor cultivation

**Strain**	**Max. temp robot screening**	**Max. temp incubator screening**	**Max. temp bioreactor**	**Confirmed mutations**
wt	44°C	42.3°C	<43°C	None
MutIV-mix	45.7°C	48°C	45.0°C	Various
MutIV-mix-2			45.8°C	Various
MutIV-C5K		44.5°C	44.9°C	10
MutIV-A1	44.9°C		45.0°C	7
MutIV-A6	45.1°C		45.0°C	2
MutIV-H2	45.4°C		45.2°C	3
MutIV-H12	45.2°C		45.0°C	7

The goal of the screening was to identify strains with notably increased thermal tolerance over the wild-type. This was achieved in both screening setups for the selected strains, though the wild-type control did outperform many other strains included in the screening (data not shown). Meanwhile the previously generated thermo-tolerant strain-mix, out of which all strains were isolated (mutIV-mix), was also included in the screening as a positive control and consistently demonstrated the highest thermal tolerance. The thermal tolerances of the strains selected for bioreactor validation and subsequent sequencing (C5K, A1, A6, H2, and H12) while considerably higher than the wild type's, but did not quite match mutIV-mix. The mix also appears to have a somewhat more robust growth rate over a range of temperatures while the single clones have a sharper optimal range. This effect is especially noticeable in the incubator screening (Figure 1).

The characterization of the selected strains in the target bioreactors yielded maximally tolerated temperatures between 44.9 and 45.1°C. The determined tolerances are very close to the 45°C the strain mix (mutIV-mix) was growing stably at, at the point they were isolated.

### Mutations in selected thermo tolerant stains

The total DNA from the five individual strains selected by the screening process, as well as a wild-type control, were each sequenced with NGS technology. The sequencing coverage for all sequencings was sufficiently high (wt-control: 41-fold, C5K: 28-fold, A1: 24-fold, A6: 20-fold, H2: 18-fold, H12: 32-fold) and the average raw accuracy across each individual base position in a read was well above 99% with a median read lenght between 141 bp and 226 bp (wild-type control: 203 bp, C5K 218 bp, A1 216 bp, A6 141 bp, H2 226 bp, H12 221 bp). As expected the wild-type control did not show any new mutations compared to the parental wild type, even after the screening process.

For the thermo-tolerant strains various mutations were identified. To exclude false positives introduced by the sequencing technology or the mapping algorithm used, results were confirmed with Sanger sequencing. Table 2 shows a matrix of the true positive mutations which were identified in the thermotolerant strains by NGS and validated with Sanger sequencing. As suspected, all tested indels in homopolymer regions identified with NGS were artifacts of the sequencing technologies (discussed in background) and were confirmed as false positives through Sanger sequencing.

**Table 2:** Matrix of true-positive mutations (marked with X) identified in the various thermo tolerant strains

**Position**	**Mutation type**	**Gene**	**WT**	**Mutant**	**Strains**				
					**C5K**	**A1**	**A6**	**H2**	**H12**
96437	AA change	pnp	gAg	gGg	X*				
98129	AA change	pnp	aTg	aCg	X*	X			X*
195523	AA change	slr1098	gGc	gTc	X*				
494155	AA change	PilJ	Cgg	Tgg			X	X*	X*
1103495	AA change	pyk2	ttC	ttA				X*	
1103586	AA change	pyk2	gGa	gAa	X	X			X*
1133812	Intergenic	Intergenic	G	T		X			
1436521	Frameshift	cya1	-	GCAA		X			
2260225	Intergenic	Intergenic	-	A	X	X			X*
2466798	AA change	clpC	gTg	gCg					X*
2466808	AA change	clpC	Cgt	Tgt	X*	X			
2467723	AA change	clpC	Gac	Tac	X*				
2468031	AA change	clpC	gTt	gCt	X*				
2579829	AA change	sll0064	Ttg	Atg	X*				X*
2717197	Frameshift	pyrR	-	TAATTAACTCCAC	X	X			
3371838	AA change	SigF	Cgt	Tgt			X	X*	X

Mutations marked with *indicate mixed reads in Sanger (& NGS), where the wt variant can also still be detected.

### Prolonged In vivo selection within the strain mix

The various strains used for screening were isolated out of mutIV-mix, a mix of many strains generated through random mutagenesis and *in vivo* selection growing stably at 45°C. Continuing selection on mutIV-mix for one year after isolation of the monoclonal strains (by keeping the selective pressure high and raising the temperature whenever stable growth was achieved) further raised the maximally tolerated temperature with stable growth up to 45.8°C (see Table 1). This new strain-mix with higher thermal tolerance was dubbed mutIV-mix-2. It was sequenced with NGS to validate mutations identified in the monoclonal strains and to gauge the distribution of mutations after prolonged selection. To asses the distribution in of the mutations within the mix a high coverage was achieved (123.5-fold); the average raw accuracy across each individual base position in a read was at 99% with a median read length of 188 bp. The results are listed in Tables 3 and 4.

**Table 3:** **Mutations identified in mutIV-mix-2 in genes also identified in the monoclonal strains (Table **2**)**

**Position**	**Mutation type**	**Gene**	**WT**	**Mutant**	**Percentage of reads**
98129*	AA change	pnp	aTg	aCg	100
194955	Frameshift	slr1098	-	T	11.70
195111	AA change	slr1098	Agt	Cgt	23.7
1103495*	AA change	pyk2	ttC	ttA	0.68
1103586*	AA change	pyk2	gGa	gAa	94.4
1133812*	Intergenic	Intergenic	G	T	51.4
1435867	Frameshift	cya1	ATGGTCGACG	-	41.3
2260225*	Intergenic	Intergenic	-	A	100
2466798*	AA change	clpC	gTg	gCg	45.5
2466808*	AA change	clpC	Cgt	Tgt	8.16
2466882	AA change	clpC	gTa	gCa	10
2467723*	AA change	clpC	Gac	Tac	4.65
2468031*	AA change	clpC	gTt	gCt	20.5
2579430	Frameshift	sll0064	-	CCCTG	61
2717197	Frameshift	pyrR	AAGGTTAA	GTGGCTTTAA…	10.9
2717187	Frameshift	pyrR	-	TTAACTCCACTAA	70.4

Positions marked with a *correspond to identical mutation positions for monoclonal strains.

**Table 4:** Mutations in genes only identified in mutIV-mix-2 and not previously observed in a monoclonal strain

**Position**	**Mutation type**	**Gene**	**WT**	**Mutant**	**Percentage of reads**
57479	AA change	glgA	gCc	gTc	22
853615	AA change	rpoC2	gCc	gTc	38
2499596	AA change	recG	gCc	gTc	70
2850065	AA change	hemH	Gat	Tat	22
3169828	AA change	cmpA	Ggt	Agt	54
3553027	Intergenic	Intergenic	A	G	16

### Identified genes

The gene that stands out the most from those identified (Tables 2 and 3 and 4) is ***clpC***, an ATP-dependent Clp protease ATPase subunit. In total five distinct mutations where identified in this gene. Four within the monoclonal strains, and an additional one in the strain mix. No single strain appears to contain all of the mutations however. C5K contains the most, with three distinct mutations, one of which A1 also carries, while H12 has another unique mutation (Table 2). Proteases are responsible for digesting misfolded proteins, and are typically up-regulated as part of the heat shock response. In *Synechocystis clpC* has been linked to various stress responses, its expression has been shown to be increased after heat shock [16], high-salt acclimation [25] and UV or high-light irradiation [26]. Four of the mutations found in *clpC* (positions 2466798, 2466808, 2466882 & 2467723 - present in H12/mutIV-mix-2, A1/C5K/mutIV-mix-2, mutIV-mix-2, and C5K/mutIV-mix-2 respectively) are located between the known domains of the protein. Of these, the Mutation at position 2466798 is by far the most common in mutIV-mix-2, being represented in 45% of all reads. The last mutation (position 2468031; in C5K/mutIV-mix-2) is located in the first ATPase (ATP binding) site of the protein and is the second most common in mutIV-mix-2.

A second gene presenting more than one mutation is ***pnp***, a polyribonucleotide nucleotidyltransferase. It has one amino acid (AA) change in A1, H12, C5K & mutIV-mix-2 (position 98129) and an additional one in C5K (position 96437). Though in C5K both mutations again seem to be not fully segregated. In mutIV-mix-2 the first mutation was found in all reads, while the second was not detectable. *Pnp* is part of the degradosome, which is involved in the processing of ribosomal RNA and the degradation of messenger RNA. The expression of *pnp* has previously been reported to be up-regulated in *Synechocystis* in acid stressed [27] and cold acclimated [28] cultures. The first identified mutation (position 96437; C5K) lies in the S1-like superfamily region, which contains the RNA binding sites, while the second (position 98129; C5K, A1, H12, C5K & mutIV-mix-2) is located within the RNAse domain of the protein.

Another very interesting mutation was identified is the amino acid exchange in ***sigF***, a group 3 RNA polymerase sigma factor. *SigF* has been shown to affect various processes in *Synechocystis*. It is needed for high-salt and high-light tolerances [29] and for the expression of thick pili [30,31] and motility [29,31,32]. The identified mutation occurred in the region-4 domain, which is the region of Sigma-70 proteins containing the DNA binding sites.

The same three strains (A6, H12 & H2) with a mutation in *SigF* also showed an amino acid exchange in ***pilJ***, a chemotaxis protein required for thick pili and motility [33]. The identified mutation lies within the signaling (receptor) domain of the protein.

The final gene showing more than one mutation (positions 1103495 & 1103586) is ***pyk2***, the gene coding for pyruvate kinase 2, which is essential for glycolysis [34]. No strain contains both mutations; with the exception of A6, all contain either one or the other. In mutIV-mix-2 the first mutation is in 94.4% of the reads, while the second is barely measurable. Both mutations are located within the conserved domain of the protein.

Additionally an intergenic mutation at position **2260225** was found in C5K, A1, H12 as well as mutIV-mix-2. It is upstream of ***nlpD***, a putative peptidase, and thus might affect its expression. In *E. coli nlpD* has functions in cell wall formation and maintenance [35], and has been shown to be required for cell separation (peptidoglycan degradation) [36,37].

Both the A1 and C5K strains, presented a mutation in ***pyrR***, an uracil phosphoribosyltransferase. In mutIV-mix-2 two separate frameshift mutations were detected in *pyrR*, one insertion at position 2717183 (in 10.9% of reads) and a second one at position 271787 (in 70.4% of reads). *PyrR* catalyzes conversion of uracil back to uridine 5'-monophosphate, and thereby allows its use for new RNA synthesis without de novo synthesis. The mutation in A1 and C5K inserted a repeat sequence (TAATTAACTCCA) into the end of the gene, and thus changed the end of its amino acid sequence from VELIKG to WS, shortening the 178 AA long protein by four amino acids. The mutations detected in mutIV-mix-2 also changed the terminal part of the protein. The more common mutation from KG to NSTN, the other removes a stop-codon changing the last 13 AA and adding 40 new AA (STQNAEFFCHWFSILSAAFWCVLDFWRLSDPTGG AGIPLFRPGHRGDRLKKTG). The *pyrR* multi-domain and its active sites are not affected by any of the mutation.

Two distinct frameshift mutations introducing a stop codon were detected in the gene ***cya1***, the major class III adenylate cyclase in *Synechocystis*: An insertion in the the strain A1, and a deletion in 41.3% of mutIV-mix-2 reads. The mutation in A1 truncates the 337 AA long protein by 37 AA in length and changes the last eight AA (to SNCSAAHH). The mutation in mutIV-mix-2 reduces the length by 262 AA and changes the last AA.

In the strains C5K and H12 a not fully segregated mutation causing an AA exchange, was found in the active domain of ***sll0064***, a putative polar amino acid transport system substrate-binding protein located in the periplasm. In mutIV-mix-2 a different mutation causing a frameshift leading to a truncation of 10 AA outside of the active domain was detected.

Three distinct mutations where detected in the hypothetical protein ***slr1098***. Two AA exchanges (C5K & 11.7% of mutIV-mix-2) and one frameshift (23.7% of mutIV-mix-2). The frameshift detected in mutIV-mix-2 inserts a stop codon early in the sequence, reducing the protein to just 38AA.

Additionally a base substitution was also identified on position 1133812 in the strain A1 and in 51.9% of mutIV-mix-2 reads. The mutation upstream of the hypothetical proteins ***sll0875 & slr0876***.

A few mutations were only fond in mutIV-mix-2 but not in any of the monoclonal strains (Table 4). These include mutations the genes ***recG***, ***cmpA***, ***rpoC2***, ***glgA*** and ***hemH*** as well as a mutation at position 3553027 (upstream of ***fur & slr0596***).

The most prevalent of these are the mutations in ***recG*** (70% of reads) and in ***cmpA*** (54% of reads). *RecG* is involved in DNA repair over homolog recombination [38]. The mutation leads to a AA change in the nucleotide binding region of the HELICc-Domain. *CmpA* is part of the cyanobacterial carbon concentrating mechanism (CCM) and specifically binds bicarbonate [39]. The mutation leads to an AA exchange in the active domain (NMT1_2; TauA multidomain).

## Discussion

### Temperature tolerances

We were able to identify strains with superior characteristics than the wild type by two different screening approaches. The modified Ratkowsky-Equation used for data fitting, allowed for good visualization and easier comparison of the data. The maximally tolerated temperatures determined during the screening (see Table 1) however varied somewhat from those seen in 1 l photobioreactor cultivation. The actually tolerated temperature is expected to vary to some degree depending on other cultivation conditions, such as, light, aeration, and cultivation vessel shape. Therefore selection and validation should be performed in the target cultivation system if possible. In our case the whole approach is based on the conditions in the 1 l bioreactor, (used for selection and validation) which are a good approximation for outdoor bioreactor conditions.

The variation between cultivation systems is especially high for the incubator screening: The maximally tolerated temperature of the C5K strain determined in this system for example, is quite a bit lower (0.4°C) than the one determined in the photobioreactor validation. Meanwhile for the strain mix (mutIV-mix) a far higher thermal tolerance was determined in the incubator screening compared to the photobioreactor validation (0.7°C; see Table 1). In comparison the maximally tolerated temperatures determined in the high throughput robot system were much more in line with bioreactor cultivation data, demonstrating the superiority of this system, not only in throughput and handling, but also in the quality of generated data.

During screening, the strain mix (mutIV-mix-1) also showed a more robust growth over a range of temperatures, which is to be expected as the measured curve results from the average of a number of strains with differing optima. Overall validation of the selected strains in 1 l photobioreactors showed them to be as thermo tolerant (~45°C) as the strain mix they were isolated out of, underpinning the validity of the approach.

Prolonged cultivation of the starting mix (mutIV-mix) over one year under selective conditions in the bioreactor further increased temperature tolerance by an additional 0.8°C (mutIV-mix-2). We assume that no new mutations took place during the cultivation of mutIV-mix-2, after the isolation of the monoclonal strains described above. This is likely to be the case due to the relatively short time frame, lack of additional rounds of mutagenesis and low natural mutation rate. Thus this further increase in thermo-tolerance can probably be attributed to further selection and enrichment of higher performing strains already present within mutIV-mix. Those better strains were not identified with the screening because of the relatively low throughput of the systems used with only 27 candidate strains in the incubator screening and 81 strains in the robot screening. Unlike the incubator screening, the robot screening platform however is highly scalable and could be expanded to create a true high throughput culturing and screening system for phototrophic organisms.

It is also of interest that so few of the strains isolated from the mix outperformed the wild type at all. One would expect all strains contained within the mix to have a thermal tolerance to at least the temperature they were cultured at within the mix. Otherwise they should have been out-competed by other strains, however this does not seem to have been the case. There are several possible explanations for this behavior. It could be, that many strains within the mix (perhaps even those with the highest thermal tolerance) are deficient in other aspects. They could be unable to grow on agar plates, or they could lack the ability to grow monoclonally in any medium (for example because they no longer produce certain vitamins which are readily available in the mix culture or other synergistic effects). They could also have become dependent on specific factors within the photobioreactor cultivation system for fast growth, such as the controlled pH-range, the strong mixing, or the relatively high cell density, where other cells provide shading.

### Genetic mutations

The reference wild type appears to have amassed various mutations over time compared to the genome published in 1996. Most are SNPs which may or may not affect the phenotype, while the larger genomic rearrangements appear to be due to mobile transposon elements (ISY203b, ISY203e, ISY203g and ISY203j respectively). Additionally, it should be noted, that the plasmids pCC5.2 and pCB2.4 could not be detected with NGS technology. This has previously been reported, and appears to be an artifact of the technologies used, as both plasmids are detectable by PCR [40]. The genetic drift of the parental wild-type strain during extended laboratory cultivation is not a main topic for this work however, and was not examined in detail. Variation found in our parental wild-type strain compared to the Kazusa strain sequenced in 1996 [19] were not validated with Sanger sequencing. However a re-sequencing of a wt clone after screening (as well as the sequenced enhanced strains) presented the same identified variations.

The lack of new mutations in the wild type, even after selective screening, shows a relative stability in the genome, and demonstrates the need for a mutagen to obtain advantageous mutations in an acceptable time frame.

In the enhanced strains various (though overall surprisingly few) mutations were found. As expected some mutations identified by NGS in homopolymer regions turned out to be false positives upon Sanger validation and were excluded from the analysis. However, the remaining mutations identified are likely to offer some form of increased fitness at higher temperatures, as disadvantageous or neutral mutations are not expected to propagate through the many genomes in *Synechocystis* and are thus likely to be lost after a couple of generations or at the very least not be detectable by NGS or Sanger sequencing.

Advantageous mutations in contrast are expected to multiply within *Synechocystis* genomes during the long selection process, while separating from neutral or disadvantageous mutations which might have generated at the same time. This, together with the relatively low mutagen dosages used (which were specifically selected for optimal point mutation rate), explains the low number of specific hits, and lack of noise from other mutations within the sequencing data.

Overall, the mutation distribution identified in the monoclonal strains selected via the screening procedure (see Table 2) is not easily explained trough linear ancestry between strains. At first glance it appears that within the 5 strains analyzed there are two distinct lineages, one containing the strains C5K & A1 and the other containing A6, H2 & H12, (possibly with A6 as a common ancestor). A6, H2 & H12 also showed very similar growth rates during the screening (see Figure 2). However some of the strains share mutations across these two putative lineages. For example distinct mutations in *pyk2* and *pnp* are found in C5K, A1 & H12 and another in *sll0064* is found in C5K and H12. This indicates that at least some of the mutations must have occurred independently at the same position in various strains. This validates the significance and importance of these specific mutations to achieve a higher thermal tolerance in *Synechocystis*.

All mutations identified are assumed to have at least some degree of effect on the strains temperature tolerances. Speculation on the possible mechanism for these mutations to facilitate a higher temperature tolerance is presented below.

Five distinct mutations were identified in the gene ***clpC***. It is very likely that these affect the activity of the protein, either making it better able to cope with the increased demand or helping it to keep its normal activity up at increased temperature. It is also interesting to note how the mutations are distributed. The monoclonal strain containing more than one mutation in *clpC* (C5K) always had mixed sequencing signals, indicating that they are not fully segregated through all of the genomes. The sequencing of mutIV-mix-2 also show various mutations, with differing percentages. These results indicate that the wild-type copy might still represented in some of the genomes, and that there are various differing mutant copies of the gene, each with one or more of the base substitutions present. Overall the results point towards each point-mutation excluding others within the same gene copy, rather than a combination of various mutations within a single copy. For the first three mutation positions, which have less than 100 bp distance from each other and can thus be covered by a single sequencing-read, no read showed more than one of the mutations simultaneously. This implies that the various mutations are not complementary but instead reach a similar phenothypic outcome through different means. The most common mutation in mutIV-mix-2 (at position 2466798) might provide the highest fitness advantage at increased temperatures, however this effect does not appear to be high enough to fully out-compete all other mutations even after one year of stringent selection.

Both mutations detected in ***pnp*** again might increase its activity at higher temperatures. The position of the first mutation within the binding site could also indicate a change the enzymes specificity towards certain RNAs, thus changing overall protein expression.

In mutIV-mix-2 however only the second mutation which lies within the RNAse domain was found, indicating that this mutation is superior to the other and thus propagated across all strains during the prolonged cultivation. Together with the intergenic mutation at position 2260225, it is the only mutation found in all reads of mutIV-mix-2, and thus appears to confer a large fitness advantage at increased temperature. A propagation of this mutation across all the strains merely through early formation can be excluded, as at the time of single clone isolation the monoclonal strains A6 and H2 did not contain it.

The identified mutation in ***sigF*** occurred within the DNA binding sites and thus probably affects its binding characteristics. The effect of altered binding fro a sigma factor can potentially be very far reaching (and unfortunately unpredictable) by affecting the expression of many proteins. This characteristic has previously been exploited to significantly increase ethanol tolerance in yeast, in a method called global transcription machinery engineering (gTME), where a library of random mutagenized transcription factors is screened for their phenotypic effects [41]. Two out of three monoclonal strains identified carrying this mutation (A6, H2) show only one or two additional mutations (both in *pilJ*, and H2 also one in *pyk2*). These strains do however have a strongly increased thermal tolerance; comparable to that of strains with far more mutations (see Table 1), indicating the strong phenotypic effects of this single mutation.

*SigF* was one of only two genes for which, while they presented a mutation in some monoclonal strains, none could be detected in mutIV-mix-2 (the other being *pilJ*). This implies that there is a limit for the maximally tolerated temperature reachable with this mutation, as no strain form the mix growing at higher temperatures contains it. Or it might also lead to negative consequences due to deregulation of other genes, giving other mutations conferring increased thermal tolerance an advantage in selection.

The postion of the ***pilJ*** *m*utation indicates it might affect the genes function. It could also have a synergistic effect with the mutation in identified *SigF* as these mutations were only ever identified in conjunction in the same three strains (A6, H12 & H2). Either way, as discussed above, both mutations were not detectable in any reads of mutIV-mix-2, demonstrating that they might only lead to a local maximum concerning temperature tolerance in *Synechocystis*.

Two mutations were detected in ***pyk2***. Since pyruvate kinase is essential for glycolysis, and thus needed for the survival of the organism, a knockout or strong inhibition of *pyk2* can be excluded. Both mutations are within the conserved domain of *pyk2* and could indicate that in *Synechocystis* the reduced activity of the pyruvate kinase at higher temperatures could be one of the bottlenecks inhibiting growth, which is alleviated by either of the identified mutations. The distribution in mutIV-mix-2 indicates that the second mutation seems to be less effective at increasing temperature tolerance, as it has been practically completely displaced by the first.

The mutation upstream of ***nlpD*** could lead to increased or reduced expression of the protein, thus it might affect cell wall composition or aggregation behavior of the cells, making them more likely to stick together, thus protecting the inner cells from the increased temperature. Within the screening platform the strains carrying this mutation where among those where a higher adhesion towards the well walls was observed (data not shown). Together with the previously discussed mutation in *pnp* this is the only mutation identified in all reads of mutIV-mix-2. This indicates that all (or at least almost all) strains contain this mutation in all genome copies indicating a high fitness advantage at increased temperature. A large distribution of this mutation due to early formation can again be excluded, as not all monoclonal strains contained it.

Even though no mutation in ***pyrR*** was within the conserved domain, they could still affect protein activity. Especially when considered in conjunction with the previously discussed mutations in *pnp*, this could indicate an increased activity for for both Enzymes because of a higher turnover of mRNA. In mutIV-mix-2 in total 81.3% of the reads showed a mutation in pyrR, which demonstrates a high importance for increased thermal tolerance.

For ***cya1*** even the more conservative mutation identified, already removes some of the catalytic sites in the conserved CyaA domain of the protein, and thus probably inhibit its function. Adenylate cyclases are important regulators for various cell processes. They generate cAMP, which is a second messenger that participates in a wide variety of signal transduction systems. In *Synechocystis* a *cya1* knockout has been shown to be impaired in motility [42,43]. The effects of a frameshift mutation in *cya1* could be very far reaching however and affect cellular regulation in many ways beyond its motility. In mutIV-mix-2 the mutation from A6 was no longer detectable, instead a mutation was detected which is even more likely to inhibit protein function. Its spread across 41.3% of the reads indicates that this inhibition has a notable fitness advantage at higher temperatures.

For ***sll0064***, ***slr1098*** as well the mutation upstream of the hypothetical proteins ***sll0875 & slr0876*** it is difficult to speculate upon the possible effects, as the full function of the proteins in *Synechocystis* is still unknown.

While sll0064 is classified as a putative amino acid transporter, *sll0875 & slr0876* have unknown functions and have either very low identity to known proteins, or are only similar to other proteins of unknown function, making it impossible to speculate of the discrete effects. *For slr1098* ortholog search results however seem to indicate it contains a HEAT domain, and thus might be involved in intracellular transport. [44,45]. The early insertion of a Stop-codon in *slr1098* definitely constitutes a knock out.

For *sll0064 and sll0875*/*slr0876* the incidence of the mutations seams to have increased after the prolonged section process (from 2/5 and 1/5 of monoclonal strains, to 61% and 51.9% of all reads in mutIV-mix-2 respectively), indicating an advantage at higher temperatures.

Meanwhile the mutations in *slr1098* do not appear to be as important for high temperature tolerance as only one of the monoclonal strains, and only a low percentage of mutIV-mix-2 reads, included one. The effects should also not be fully excluded however as three distinct mutations propagated across the stains/genomes.

For the mutations only identified in mutIV-mix-2 with very few reads (**rpoC2**, **glgA**, **hemH**, **fur & slr0596**) it is impossible to determine if these are very rare true positives, or if it is a sequencing error (some of which are statistically expected to be identical in more than one read). A validation via PCR and Sanger-sequencing can also not be performed as the competitive PCR process would confer an advantage the more common variant.

For the mutations identified in ***recG*** and ***cmpA*** however, the large number of reads identifies them as true positives. As no further mutagenesis was performed after the isolation of the monoclonal strains it is very likely that these two mutations were already present in the mix before the prolonged selection, albeit probably in lower numbers (less strains).

The mutations in *recG* and *cmpA* are both within the active (/binding) sites and thus might affects their binding characteristics (to nucleotides or bicarbonate respectively). Again these mutations could increase the activity or maintaining the activity at higher temperatures. The mutation at CmpA is especially interesting as phototrophic organisms typically require a more specific RuBisCo at higher temperatures to compensate for the different concentration ratios of dissolved O_2_/CO_2_[46]. Cyanobacteria instead increase the local CO_2_ concentration via the CCM. Thus this mutation might increase the overall efficacy of the CCM.

## Conclusions

Overall the approach of using random mutagenesis, followed by *in vivo* selection, selective screening, and then genetic sequencing was found to be very promising. Many interesting mutations could be identified which increase the thermal tolerance in *Synechocystis*. With only two exceptions all genes containing mutations in the isolated monoclonal strains were confirmed by sequencing the strain mix after prolonged selection, further validating the results. Some mutations in genes (such as *pyk2* – coding for pyruvate kinase 2) are not obvious candidates for temperature tolerance increases, and are thus very unlikely to have been identified by other approaches.

With quite a few genes now identified as being involved in the temperature tolerance of the strains, further strains could be selected at random and then analyzed at low cost with a PCR panel and Sanger sequencing. Thereby providing data to analyze the distribution of the mutations in the strain mix.

Further studies beyond the scope of this work should isolate the individual effect on the temperature tolerance of each mutation by transforming it into a wt strain and characterizing it.

Additionally gene expression profiling of some of the strains, or of a wild type with specific mutations introduced, would probably also yield very interesting results. This is of special interest for mutations such as the one found in sigma factor F (*sigF*) which could affect the expression of many different genes. Sigma factor F is now also an obvious candidate (over the other nine sigma factors present in *Synechocystis*) for global transcription machinery engineering (gTME).

The underlying functional differences resulting from the mutated genes could now also be characterized (e.g. *pyk2* activity over temperature) and be compared to the wild-type protein.

The function of the hypothetical proteins found to be altered should also be investigated, by creating respective knockout strains in *Synechocystis*.

Introducing various identified mutations into a single strain and analyzing the resulting phenotype would also be of considerable interest. It is quite likely that some combinations could lead to strains with even higher temperature tolerances, which could then be used as a starting point for further enhancements, either with targeted methods on known genes, or with new rounds of random mutagenesis.

## Methods

### Organisms

•*Synechocystis* sp. PCC 6803 salt adapted wild type, provided by Algenol Biofuels Germany GmbH

•*Synechocystis* sp. PCC 6803 - mutIV, mix of various thermo-tolerant strains [6]

### Media

All strains were cultivated in sterile mBG11 Media (63 g/l Instant Ocean®, 17.65 mM NaNO3, 0.18 mM K_2_HPO_4_, 0.03 mM Citric acid, 0.003 mM EDTA (disodium magnesium), 0.19 mM Na_2_CO_3_, 0.03 mM Ferric ammonium citrate and trace metals).

### Generation of thermo-tolerant mutant pool

The starting pool of mutants (mutIV-mix) was generated through four rounds of mutagenesis (2 X UV at 50 J/m^2^ and 2 X MMS at 0.1v%/1v% for 1 min). After each mutagenic treatment cultures were kept under highly selective conditions, thereby performing an *in vivo* selection for cells with increased temperature tolerance. The generated pool was able to stably grow at 45°C while the wild type would not tolerate 43°C. For details please see [6].

### Monoclonal strain isolation

Monoclonal strains were isolated from the previously generated mix of thermo-tolerant strains (cultivated at 45°C at the time of isolation), by plating 500 cells on BG11-1%-Agar plates. Plates were cultivated at a constant temperature of 40°C to keep selection pressure on thermal tolerance. After about 2 weeks single clones were picked with a sterile loop and transferred into 1.5 ml mBG11 in 96-Deepwell plates (DWPs) or 15 ml mBG11 in T25 cell culture flasks for temperature tolerance screening.

### Temperature tolerance screening

Isolated monoclonal strains were grown competitively in either T25 cell culture flasks in an Infors Minitron Incubator (27 strains) or in a 96-Deepwell plate (81 strains) in a custom build automated cultivation chamber integrated into a liquid handling robot.

In both setups, the starting thermo-tolerant strain-mix (4 wells for robot screening), as well as a wild-type clone (6 wells for robot screening) were also cultivated as positive and negative controls respectively. In the DWPs 4 wells contained sterile media, and were used as a control for the detection of potential cross contaminations.

In the incubator for the T25 cell culture flasks night and day cycles were set to 12 h each. During the day phase, illumination was set to 19 μE/m^2^, 55 rpm orbital shaking, aeration with 0.5% CO2 in the chamber, and temperature starting at 28°C up to 47°C at the end of the screening. Night temperature was set to 25°C and 25 rpm shaking. OD750 was measured daily with a Shimadzu UV-1800 photometer.

The cultivation chamber is integrated into a Tecan RSP150 robot and can be fully handled by the robots object manipulation arm. Cultures were kept in suspension by orbital shaking at 750 rpm. To avoid cell aggregates and cell adhesion to walls of the DWP, three glass beads (1 mm ø) were added to each well. The atmosphere was set to 2% CO_2_. The setup allows automated detection of OD (with Tecan Genios Pro Plate reader). Cultures were temperated through a heated metal plate on which the DWP rests. Temperature (measured in a reference well) is PID controlled and has a maximum measured deviation of 0.2°C from the set value. Day temperature and light were increased stepwise from 30°C and 3 μE/(m^2^*s) (first 3 days after inoculation) up to 47°C and 85 μE/(m^2^*s). Night temperature was set to 26°C.

For both systems day-temperatures were raised in 0.5°C increments until 35°C (approx. optimal growth rate of the wt). Depending on behavior of the mix of thermotolerant strains (mutIV-mix), which was cultivated as control, the temperature was raised in maximum increments of 0.2 or 0.1°C per day. Each temperature for which a growth rate was determined was held at least until a minimum of three OD values could be measured (3–7 days).

### Analysis of screening data

The growth rate (μ) was calculated for each strain at any given temperature by comparing the OD_750_ at the time point 0 (X_0_) with OD_750_ after time t (X_t_):

$$\mu =1n\frac{X_{t}}{X_{0}}\cdot \frac{1}{t}$$

To allow an easier comparison of data, and a faster identification of performing strains, the behavior of the strains over temperature was fitted using a modified Ratkowsky-Equation [47,48]:

$$\mu ={\left(b\left(T-T_{\text{min}}\right)\right)}^{2}\;\cdot \left(1-e^{c\left(T-T_{\text{max}}\right)}\right)$$

where T_min_ (°C) is the minimum, while T_max_ (°C) is the maximum temperature allowing growth.

The Ratkowsky parameters b and c were calculated according to the following formulas:

$$b=\frac{\Sigma \left(X_{i}-\overset{-}{X}\right)\;\left(y_{i}-\overset{-}{y}\right)}{\Sigma {\left(X_{i}-\overset{-}{X}\right)}^{2}}$$

$$c\left(T-T_{\text{max}}\right)=\mathrm{lo}g1-\;\frac{\sqrt{\mu }}{b\left(T-T_{\text{max}}\right)}$$

where x_i_ are the calculated growth rates with their mean $\overset{-}{X}$, while y_i_ and $\overset{-}{y}$ represent the respective temperatures and mean temperatures.

Minimum growth temperature was not measured, but instead determined using the first Ratkowsky-Equation:

$$\sqrt{\mu =b\cdot \left(T-T_{\text{min}}\right)}$$

Only data in the linear range, below the optimal growth rate, was used for this calculation.

### Validation of thermal tolerance in bioreactors

The five strains identified in the screening as the most thermal-tolerant were validated through cultivation in a 1 l photobioreactor before being sequenced. The pH value was controlled through the periodic addition of 10% CO2/air mixture. During a 12 h daylight phase, pH was kept at 7.3+/−0.05, light at about 125 μE/m^2^*s (170 from one side and 80 μE/(m^2^*s) from the other) and the culture was actively temperated. During the night phase cultures were bubbled with 10 ml/min air, and (passively) cooled down to 23–26°C. Cultures were diluted with fresh media so that they stayed in the logarithmic growth phase, within an OD_750_ between 1 and 2 (measured with a Shimadzu UV-1800 photometer). Cultivation temperature was increased stepwise until the maximum tolerated temperature was found. A culture was considered to be growing stably at a given temperature, if it grew consistently at that temperature for at least two weeks. The maximum tolerated temperature was determined by raising the temperature in in 0.1°C increments until stable growth (as defined above) was no longer possible. The previous lower temperature still allowing stable growth is the maximum tolerated temperature.

### Prolonged in vivo selection within thermo-tolerant mix

Over the time span of one year, the strain-mix (mutIV), was continually cultivated under selective pressure in a bioreactor with the conditions described above. The temperature was raised in 0.1°C increments whenever stable growth was achieved, and lowered (allowing for recovery) if a lack of growth showed the maximum tolerated temperature was exceeded.

### DNA extraction

DNA was extracted with a novel two step protocol minimizing the amount of foreign (non *Synechocystis*) DNA based on the protocol of Franche and Damerval [49].

All samples were prepared by centrifugation at low g to reduce the content of potential heterotrophic contaminants. 1 ml of culture (OD_750_ = 1) was centrifuged three times at 500 g for 10 min, the supernatant discarded and the resulting pellet resuspended in sterile media.

For the first lysis step the sample was centrifuged at 13,000 g for 10 min. The pellet was washed twice in TE buffer (10 mM TRIS, 1 mM EDTA, pH 8) and then resuspended in 500 μl TES buffer (50 mM Tris, 100 mM EDTA, 25% Sucrose, pH 8) for an osmotic shock and incubated 60 min at 4°C. 500 μg Lysozyme was added before 240 min incubation at 37°C. The sample was then centrifuged at 13000 g for 10 min. The pellet was washed once with Tris buffer and then resuspended in Tris with two units of DNaseI and incubated for 30 min at 37°C. The sample was then centrifuged, washed and resuspended in TE buffer.

A second, stronger, lysis was then performed by repeating the above steps up to and including the addition of Lysozyme, incubating for 30 min at 37°C before adding 50 μl 20% SDS and 8.4 μl ProteaseK (600 mAU/ml) and then incubating overnight (12-16 h). DNA was extracted by adding one volume of phenol/chloroform, vortexing and phase separation by centrifugation at 13000 g for 10 min using PhaseLockGel tubes. The aqueous phase was transferred to a new tube and one volume of chloroform/isoamylalcohol (24:1) was added. After vortexing and centrifugation with the parameters above the aqueous phase was transferred and containing DNA was precipitated using 0.7 volumes of isopropanol and centrifugation at 13000 g for 30 min and 4°C. The pellet was washed twice with 70% ethanol, then dried and resuspendend in 30 μl DEPC water.

RNA was then digested by adding 1 μl RNAseI (10 U/μl) and incubation for 30 min. Afterwards RNAseI was deactivated by heat shock (75°C, 5 min). The DNA content was measured using a Nanodrop Spectrophotometer.

### Next-geneneration-sequencing

To identify the mutations in the various strains with thermal tolerance, each of the selected five strains, as well as a wt-control (which also went through the thermal tolerance screening process), and the mutIV-mix-2 strain mixture were sequenced with the Ion Torrent PGM. As a baseline reference, an axenic wt clone, without any thermal tolerance selection or mutagenesis, was sequenced with the Roche 454 Junior Sequencer.

For the reference wt two sequencing runs were performed with DNA fragmented via nitrogen nebulization followed by end repair and library preparation. Library quality was controlled with a capillary gel electrophoresis (Agilent Bioanalyzer), and quantified via the fluorescent beads (TBS 380 Fluorometer) before performing an emulsion PCR amplification and then the shotgun sequencing. All steps were performed according to manufacturer protocols (Rapid Library Preparation Method Manual rev. March 2011; emPCR Amplification Method Manual – Lib-L re. April 2011; Sequencing Method Manual rev. June 2010).

For each screening candidate selected for sequencing and mutIV-mix-2, isolated genomic DNA was fragmented with the Biorupter followed by construction of a shotgun library using the Ion Plus Fragment Library Kit. The quality of the fragments was checked with the 2100 Bioanalyzer (DNA High Sensitivity Kit) and the quantity was assessed on the Roche LightCycler 480 (Ion Library Quantitation Kit). Template preparation and enrichment were performed automatically on the Ion OneTouch devices by means of Ion OneTouch 200 Template Kit v2 DL. Quality of the template preparation and enrichment was checked on the Qubit 2.0 Fluorometer (Ion Sphere Quality Control Kit). The enriched template was loaded to a 314 or 316 (for mutIV-mix-2) chip and sequenced on the IonTorrent PGM using the Ion PGM 200 Sequencing Kit (wt-control; two runs) or Ion PGM 300 Sequencing Kit (C5K, A1, A6, H2, H12 & mutIV-mix-2).

### Identification of mutations

Data from the NGS sequencing runs (in SFF format) was mapped against the previously sequenced reference wt [GenBank:NC_000911.1; NC_005229.1; NC_005232.1; NC_005230.1; NC_005231.1; CP003272.1; CP003270.1; L25424.1] using the software Roche gsMapper [50]. Data was then further analyzed with snpEFF [51], and various scripts (bash or Perl) especially written for the analysis; all scripts are attached as additional files, as well as available online at github [52].

First relevant lines were extracted from the generated output file for high confidence differences generated by gsMapper (454HCDiffs.txt) and the chromosome/plasmid names changed with the bash script Additional file 2.

The newly generated file was then converted with the Perl script Additional file 3 to a VCF file which is accepted as input for snpEff. For all enhanced strains, this output file was then compared to the mutations found in the starting wt strain, subtracting all mutations already present in the wild type using the Perl script Additional file 4.

SnpEff, a program to determine the effect of SNPs [51], was then run on the resulting VCF file against the available *Synechocystis* database in snpEff [snpEff:Synechocystis_PCC_6803_uid57659].

The third Perl script Additional file 5 was then used to extend the output of snpEff by adding information on the function, category, subcategory as listed in the category.csv flatfile provided by Cyanobase [53] (Additional file 6) and a link to the Cyanobase entry for mutations within specific genes. The output from this script is in a human readable format and can be opened with a spreadsheet program.

Finally mutations contained in this list were manually verified using Tablet Genome viewer [54] by looking up the mutation positions in the BAM files generated by gsMapper.

### Validation of identified mutations and effect determination

Mutations identified by the analysis of next-gen-sequencing data, were validated with Sanger sequencing (except those which were obviously false positives - such as missing bases in some reads within homopolymer regions). Selected regions were amplified by PCR (melting 30 s, 95°C; annealing 30 s, 56°C; extension 30 s, 72°C; 30 Cycles) with specific primers, purified with a PCRapace Kit (Stratec GmbH) according to manufacturer specifications, and then send to Starseq GmbH for sequencing.

The Sanger-sequencing data was visualized with finchtv [55] and the validity of the putative mutation verified.

Additionally five Indel mutations identified within homopolymer regions with the most reads showing missing bases, thus being the most likely true positives, where also verified as described above to validate the assumption of classifying them as false positives.

To better speculate on the possible effects of the identified mutations, the amino acid sequence was compared against the NCBI Conserved Domain Database (CDD). It was checked if the changes in the protein affect known domains.

### Primer design and sequences

Primers were designed with Perl Primer [56] to be 100-150 bp (fw primer) or 350-400 bp (rv primer) from the target base (or first target base for primecr pairs covering multiple mutations). Melting temperature was set to 58-62°C with a maximum difference of 2°C between primer pairs.

All primers were ordered from Thermo Fisher Scientific and are RP-HPLC purified. The full list of primers can be found in Additional file 7: Table S2.

## Competing interests

The submitted work was performed in cooperation with Algenol Biofuels Germany GmbH. Algenol Biofuels actively researches various systems to produce biofuels with cyanobacteria and has a vested interest in its success.

## Authors’ contributions

UMT conceived and designed the experiments, performed the *in vivo* selection on the mutIV-mix, the bioreactor validation of the selected strains, the PCRs for Sanger Sequencing, the 454 Sequencing runs (together with Markus Grohme), analyzed NGS and Sanger results, co-designed and established the screening platform, and wrote the manuscript. NW co-designed and established the screening platform, performed the screening of the strains, analyzed screening data, extracted DNA from target strains, and reviewed drafts of the manuscript. PF performed all Ion torrent sequencing runs, and reviewed drafts of the manuscript. UD assisted with experimental design, helped with cyanobacteria biotechnology know-how and reviewed drafts of the manuscript. MF supervised the research, provided laboratory facilities and contributed to the writing of the manuscript. All authors read and approved the final manuscript.

## Supplementary Material

Additional file 1: Table S1 List of SNP mutations identified with NGS in the starting wt compared to the Kazusa-wt used as a reference for mapping. Click here for file

Additional file 2 **Bash script to extract relevant lines from output generated by gsMapper (454HCDiffs.txt) and to the chromosome/plasmid names changed.** Also available online at https://github.com/utillich/NGS_Scripts. Click here for file

Additional file 3 **Converts output from “Extract_454HCDiffs.sh” to a VCF file which is accepted as input for snpEff. **Also available online at https://github.com/utillich/NGS_Scripts. Click here for file

Additional file 4 **Description of data: Substracts output from one VCF file from another.** Also available online at https://github.com/utillich/NGS_Scripts. Click here for file

Additional file 5 **Parses output of snpEff (adding information on the function, category, subcategory, and a link to the Cyanobase entry) and puts out a human readable spreadsheet.** Also available online at https://github.com/utillich/NGS_Scripts. Click here for file

Additional file 6 **Slightly edited flatfile containing gene category data, provided by Cyanobase **[50]**; used as second imput for “vcf_Syn6803_identify.pl”.** Also available online at https://github.com/utillich/NGS_Scripts. Click here for file

Additional file 7: Table S2 List of primers used for PCR and Sanger validation of identified mutations. Click here for file
